# Aspirin inhibits LPS-induced macrophage activation *via* the NF-κB pathway

**DOI:** 10.1038/s41598-017-10720-4

**Published:** 2017-09-14

**Authors:** Yitong Liu, Silian Fang, Xiaoyan Li, Jie Feng, Juan Du, Lijia Guo, Yingying Su, Jian Zhou, Gang Ding, Yuxing Bai, Songling Wang, Hao Wang, Yi Liu

**Affiliations:** 1 Laboratory of Tissue Regeneration and Immunology and Department of Periodontics, Beijing Key Laboratory of Tooth Regeneration and Function Reconstruction, School of Stomatology, Capital Medical University, Beijing, P. R. China; 2 Department of Oral and Maxillofacial Surgery, the Sixth affiliated Hospital of Sun Yat-sen University, Beijing, P. R. China; 3 Department of Stomatology, Beijing Tiantan Hospital, Capital Medical University, Beijing, P. R. China; 4 Department of Stomatology, Yidu Central Hospital, Weifang Medical University, Weifang, P. R. China; 5 Department of Orthodontics, School of Stomatology, Capital Medical University, Beijing, P. R. China; 6 Salivary Gland Disease Center and Molecular Laboratory for Gene Therapy and Tooth Regeneration, School of Stomatology, Capital Medical University, Beijing, P. R. China

## Abstract

Aspirin (acetylsalicylic acid, ASA) has been shown to improve bone marrow mesenchymal stem cell-based calvarial bone regeneration by promoting osteogenesis and inhibiting osteoclastogenesis. However, it remains unknown whether aspirin influences other immune cells during bone formation. In the present study, we investigated whether ASA treatment influenced macrophage activation during the LPS inducement. We found that ASA could downregulate the expressions of iNOS and TNF-α both in mouse peritoneum macrophages and RAW264.7 cells induced by LPS *via* the IκK/IκB/NF-κB pathway and a COX_2_/PGE_2_/EP_2_/NF-κB feedback loop, without affecting the expressions of FIZZ/YM-1/ARG1 induced by IL-4. Furthermore, we created a rat mandibular bone defect model and showed that ASA treatment improved bone regeneration by inhibiting LPS-induced macrophage activation in the early stages of inflammation. Taken together, our results indicated that ASA treatment was a feasible strategy for improving bone regeneration, particularly in inflammatory conditions.

## Introduction

Mesenchymal stem cell (MSC)-based therapy has significantly improved tissue regeneration in pre-clinical models and clinical trials. Despite the recent progress in MSC-based tissue regeneration over the last few decades, the remaining major challenge we face is how to restore defective bone with high quality bone of sufficient volume to meet the needs of the body^1, 2^. During disease or injury, bone resorption and bone formation processes are complicated. Bone regeneration quality is influenced by damaged MSC functions, an inflammatory microenvironment, and the activated host immune system. We have recently shown that the host immune system has fundamental effects on the fate of transplanted MSCs during bone remodeling. Proinfammatory T cells produce tumor necrosis factor alpha (TNF-α) and interferon gamma (IFN-γ), which play critical roles^3^. Intriguingly, some studies have shown that topical administration of aspirin, or alternatively, a systemic infusion of regulatory T cells, could inhibit TNF-α and IFN-γ production, and thus, improved calvarial bone repair in rodents and minipigs^3, 4^.

Aspirin (ASA) is a commonly used non-steroidal anti-inflammatory drug (NSAID), capable of inhibiting cyclooxygenases (COX), including COX_1_ and COX_2_. Recent studies showed that ASA could improve bone mineral density in ovariectomized-induced osteoporotic conditions by regulating the balance between bone resorption and bone formation^5–12^. Compared to systemic administration of medicines, the topical administration of aspirin provided safety advantages. Our previous studies revealed that ASA treatment benefited bone healing and promoted MSC-based bone regeneration by reducing local IFN-γ and TNF-α levels^4, 7–19^. Furthermore, ASA treatment inhibited osteoclastogenesis in receptor activator of nuclear factor kappa-B ligand (RANKL)-induced RAW264.7 cells^6^, and decreased bone absorption by downregulating prostaglandin E_2_ (PGE_2_), both *in vitro* and *in vivo* ^5^. It is well-known that macrophages also play an important role in local inflammation and bone formation. However, whether ASA treatment affects macrophages remains unknown.

Macrophages have been identified as a key factor in the progression of tissue inflammation. Accumulating evidence has shown that macrophages with distinct phenotypes exert diverse effects during inflammation and tissue repair^20, 21^. For decades, we have the consensus that at least two different phenotypes of macrophages can be distinguished: the classically activated macrophages (M1) and the alternatively activated macrophages (M2). M1 macrophages can be induced by IFN-γ and lipopolysaccharide (LPS) through a classic nuclear factor kappa-B (NF-κB) signaling pathway. M2 macrophages are generated when macrophages are cultured with interleukin (IL)-4 and IL-13 in *vitro*. It is well accepted that LPS activates macrophages; LPS induces the phosphorylation of the inhibitor of kappa B (IκB) and IκB kinase (IκK), which increases P65 nuclear translocation to activate M1 macrophages. M1 macrophages are capable of producing pro-inflammatory factors, like IL-1, TNF-α, IL-6, IL-23, reactive oxygen species (ROS), nitric oxide (NO), and inducible NO-synthase (iNOS). Thus, M1 macrophages lead to inflammation, and they are predominant in the early stage of inflammation^22^. In contrast, M2 macrophages release an abundance of IL-10, anti-inflammatory cytokines, and trophic factors, such as transforming growth factor beta (TGF-β), Arginase-1 (ARG1), Retnla (FIZZ), chitinase-like 3 (Chil3, YM-1), which block the activation of T cells and macrophages; thus, M2 macrophages suppress inflammation and lead to tissue recovery processes^22, 23^. Recent years many scientists suggested macrophage polarization should not be simply described as M1/M2 macrophage due to the complication of macrophage activation^24–26^. Because macrophage could be induced to different situation by varieties of activators, we can define the macrophage polarization base on the activators such as LPS-induced macrophages or M(LPS).

This study aimed to examine the influence of ASA on macrophages under inflammatory conditions and to explore the potential molecular mechanisms underlying ASA effects. Our results showed that ASA inhibited the activation of LPS-induced macrophages through the COX_2_/PGE_2_/EP_2_/NF-κB cascade and the NF-κB pathway. Our data indicated that ASA treatment is a feasible strategy for improving bone regeneration, particularly in inflammatory conditions.

## Materials and Methods

### Chemicals and antibodies

Aspirin (over 99% purity) and dimethyl sulfoxide (DMSO) were purchased from Sigma-Aldrich (St. Louis, MO, USA). Ultrapure lipopolysaccharide *from Porphyromonas gingivalis* (LPS) was purchased from InvivoGen (San Diego, CA, USA). Antibodies specific for COX_2_, iNOS, p65 NF-κB, IκK, IκB, phosphorylated IκK (p-IκK), and p-IκB were purchased from Cell Signaling Technology (Boston, MA, USA). An anti-β-actin antibody, anti-Heat shock protein 90 (H90) antibody and anti-histone-H3 antibody were purchased from Sigma-Aldrich (St. Louis, MO, USA). The anti-MHC Class II(I-ab)-PE antibody was purchased from eBioscience (San Diego, CA, USA). The anti-F4/80-FITC antibody, FIZZ antibody, ARG1 antibody, YM-1 antibody were purchased from Abcam (Cambridge, Cambs, UK). A prostaglandin E2 receptor (EP_2_) antagonist (C23H20FNO5) was purchased from TOCRIS (Bristol, C Cnty, UK). The thioglycollate was purchased from Aobox Biotechnology (Beijing, China).

### Kits

The Mouse Peritoneal Macrophage Isolation Kit was purchased from Miltenyi Biotec (Bergisch Gladbach, Germany). An Enzyme-Linked Immunosorbent Assay (ELISA) Kit for evaluating PGE_2_ was purchased from Abcam (Cambridge, Cambs, UK). The Mouse TNF-alpha ELISA Kit was purchased from eBioscience (San Diego, CA, USA). The Nuclear and Cytoplasmic Protein Extraction Kit was purchased from TransGen Biotech (Beijing, China).

### Animals

Female C57BL/6 J mice and rats were obtained from the Institute of Animal Science of the Vital River Co., Ltd., Beijing, China. All rats were fully barrier-reared with free access to water and a regular supply of food. This study was conducted according to the approved guidelines set by the Animal Ethics Committee of the School of Stomatology, Capital Medical University (Beijing, China). All animal experiments were performed under the institutionally approved protocols for the use of animal research (Capital Medical University#2012-x-53).

### Isolation of mouse peritoneal macrophages

Six-to-eight-week-old female C57BL/6 mice were given an injection of 2 ml thioglycollate medium (4%), Five days after injection we collected all the cells obtained by peritoneal lavage. Using the Macrophage Isolation Kit (Peritoneum), macrophages were isolated by depletion of non-targget cells. The magnetically labeled non-target cells were depleted by retaining them within a MACS Column in the magnetic field of a MACS Separator, while the unlabeled macrophages were passed through the column. The separated macrophages were determined by flow cytometric analysis using anti-F4/80-FITC antibody and anti-MHC ClassII(I-ab)-PE antibody, which suggested that >90% of the cells were macrophages. The macrophage cells were cultured in the presence of RPMI medium (Invitrogen, Carlsbad, CA) supplemented with 15% heat-inactiated fetal bovine serum (FBS; Equitech-Bio, Kerrville, TX), 5% pen/Strep (Biofluids, Inc.) and 5% glutamine (Invitrogen). Cultures were incubated at 37 °C, with 5% CO_2_, in a humidified atmosphere. The cell medium was changed every 2–3 days. All cells were induced within one week after isolation.

### RAW264.7 cells culture

RAW264.7 cells (China Infrastructure of Cell Line Resources, Beijing, China) were cultured in Dulbecco’s modified Eagle’s medium (Invitrogen, Carlsbad, CA, USA) with 10% fetal bovine serum (FBS; Equitech-Bio, Kerrville, TX), 2 mM L-glutamine (Invitrogen), 100 U/ml penicillin and 100 µg/ml streptomycin (Biofluids, Inc.). Cultures were incubated at 37 °C, with 5% CO_2_, in a humidified atmosphere. The cell medium was changed every 2–3 days. Cells were passaged when they became 70% to 80% confluent.

### Induction of macrophage cells by LPS

We treated both mouse peritoneal macrophages and RAW264.7 cells with 1 μg/ml LPS for 24 hrs, and the iNOS expression levels were determined with quantitative real time PCR, Western blot, and ELISA analyses. TNF-α expression levels were evaluated with ELISA. The ratios of iNOS positive cell number in total cells were calculated.

### ASA pre-treatment inhibited LPS-induced activation of macrophages

Peritoneal macrophage cells were seeded at a density of 2 × 10^5^ cells/well in a 6-well plate after isolation, and cultured in a 37 °C in a 5% CO_2_ incubator. After treating with ASA (0, 50, 100, 150, or 200 µg/ml) for 12 hrs, the cells were induced to become LPS-induced macrophages by adding 1 μg/ml LPS, and incubating for another 24 hrs. The negative control was macrophage cells challenged with phosphate buffered saline (PBS) and DMSO. The iNOS expression levels were determined with quantitative real time PCR, Western blot. TNF-α expression levels were determined with ELISA.

### Prostaglandin receptor antagonism

We investigated the mechanism by which aspirin inhibited macrophage cells polarization to LPS-induced macrophages in RAW264.7. 2.7 nM EP_2_ antagonist, C23H20FNO5, was added at the same time as 200 µg/ml ASA. After 24 hrs, we extracted the total proteins and performed a Western blot to detect the levels of iNOS, COX_2_, IκK, p-IκK, IκB, and p-IκB proteins. The nuclear proteins were extracted separately to detect the migration of the p65 component of NF-κB into the nucleus.

### Quantitative real-time PCR analysis

After the cell treatments, total RNA was extracted from each group with Trizol reagent (Invitrogen, Carlsbad, CA, USA). We synthesized cDNA from 2 µg aliquots of total RNA, oligo(dT) (Invitrogen, Carlsbad, CA, USA), and RNaseOUTTM Recombinant Ribonuclease Inhibitor (Invitrogen, Carlsbad, CA, USA), according to the manufacturer’s protocol. Real-time PCR reactions were performed with the Power SYBR® Green PCR Master Mix (Life Technologies, Warrington, UK) and primers that targeted iNOS, COX_2_, TNF-α, YM-1, FIZZ and ARG1 (Suppl Table 1).

### Western blot analysis

Total protein was extracted with NE-PER nuclear and cytoplasmic extraction reagents (Thermo). Nuclear proteins were isolated with a mammalian nuclear and cytoplasmic protein extraction kit (TransGen Biotech). Next, 50–100 μg aliquots of protein were separated on 10% polyacrylamide-SDS gels (Pplygen) and transferred to Immobilon™-P membranes (Millipore). After blocking with TBS/5% nonfat dry milk (Pplygen) for 1 h, the membrane was incubated with antibodies against mouse PPARγ (Cell Signaling Technology), histone H3 (Bioworld), and β-actin (Sigma) overnight at 4 °C. Next, membranes were incubated with horseradish peroxidase-conjugated secondary antibodies (Pierce, Malibu, CA, USA) for 1 h at room temperature. Antibody binding was visualized with an enhanced chemiluminescence kit, according to the manufacturer’s protocols (Pierce).

### ELISA analysis

Cell culture supernatants were collected to detect the expression of TNF-α with corresponding ELISA kits. The cell lysate was extracted to detect the expression of PGE_2_ with the PGE_2_ ELISA kit. All assays were performed according to the manufacturer’s instructions.

### Immunofluorescence analysis

RAW264.7 cells (2 × 10^5^ cells/well) were seeded into 12-well plates that contained glass coverslips. Control groups were pretreated with DMSO, and the ASA groups were pretreated with 200 µg/ml ASA for 12 hrs. Then, 1 µg/ml LPS was added for another 24 hrs. Next, the glass coverslips were harvested, fixed in 4% paraformaldehyde (PFA), and blocked with 5% normal serum. Next, the cells were incubated with antibodies specific for p65 NF-κB or an isotype-matched control overnight at 4 °C. Samples were then incubated with Alexa Fluor 488 conjugated secondary antibodies (Cell Signaling Technology). Next, samples were counterstained with 4′,6-diamidino-2-phenylindole (DAPI, Vector) and mounted onto slides with glycerin. Slides were observed under a fluorescence microscope (OLYMPUS, Tokyo, Japan). Semi-quantification was performed by counting the positive signals in at least five random high-power fields. Results are expressed as percentages of the total number of DAPI-positive cells.

### Flow cytometric analysis

Peritoneal macrophage cells after isolation were determined by flow cytometric analysis. We fixed the cells in 4% paraformaldehyde (PFA), and blocked with 5% normal serum. Then the cells were incubated with anti-F4/80-FITC antibody and anti-MHC ClassII(I-ab)-PE antibody for 30 min in the dark. Cell preparations were immediately analyzed with flow cytometry (FACSCalibur, BD Bioscience).

### Establishing the rat mandibular bone defect model

Left sides of 24 rats were used for *in vivo* studies. We established mandibular bone defects (5 mm long, 2 mm wide, and 1 mm high) between the incisors and molars. All 24 rats with mandibular bone defects were randomly assigned to either the control or the aspirin group. We mixed 500 µl hydrogel with (aspirin group) or without (control group) 200 µg/ml aspirin, and 20 mg Hydroxyapatite/Tricalcium phosphate (HA/TCP) was added as a scaffold. This mixture was used to fill the mandibular bone defects. Then, the mucoperiosteum and skin incisions were closed with 4–0 absorbable sutures. At the 3^rd^, 5^th^, 14^th^, and 60^th^ day after surgery, three rats in each group were sacrificed.

### Immunohistochemistry

*In vitro* experiments macrophage cells (2 × 10^5^ cells/well) were seeded into 12-well plates that contained glass coverslips. Control groups were pretreated with DMSO, and the ASA groups were pretreated with 200 µg/ml ASA for 12 hrs. Then, 1 µg/ml LPS was added for another 24 hrs. Next, the glass coverslips were harvested, fixed in 4% paraformaldehyde (PFA). To reduce non-specific staining, all sections were incubated with 3% hydrogen peroxide for 10 min at room temperature and blocked with 10% serum for 60 min at 37 °C. Then, the pretreated sections were incubated with primary antibodies (50 µg/ml) at 4 °C overnight. Biotinylated secondary antibodies (1 µg/ml) were added, and specimens were incubated at room temperature for 1 h. Finally, the horseradish peroxidase complex was added with the diaminobenzidine substrate for visualization. After cells were counterstained, slides were observed under a light microscope (OLYMPUS, Tokyo, Japan). Semi-quantification was performed by counting the number of positively stained cells in at least five random fields.

*In vivo* we euthanized 12 rats at 3^rd^ and 5^th^ day after surgery to perform immunohistochemical examinations. The mandibular bone defects were fixed in 4% PFA. These specimens were decalcified and embedded in paraffin. Sections (6-μm thick) were cut and mounted onto slides. Unstained sections were deparaffinized and rehydrated, and antigens were retrieved with sodium citrate (pH 6.0). To reduce non-specific staining, all sections were incubated with 3% hydrogen peroxide for 10 min at room temperature and blocked with 10% serum for 60 min at 37 °C. Then, the pretreated sections were incubated with primary antibodies (50 µg/ml) at 4 °C overnight. Biotinylated secondary antibodies (1 µg/ml) were added, and specimens were incubated at room temperature for 1 h. Finally, the horseradish peroxidase complex was added with the diaminobenzidine substrate for visualization. After samples were counterstained, slides were observed under a light microscope (OLYMPUS, Tokyo, Japan). Semi-quantification was performed by counting the number of positively stained cells in at least five random fields.

### Quantitative and histological evaluations of regenerated bone

Six rats were euthanized 2 weeks after the surgery to detect the rate of healing. Another six rats were euthanized at 2 months after surgery to detect the quantity of regenerated bone with hematoxylin and eosin (H&E) staining. Briefly, bone specimens were fixed in 4% PFA. The specimens were decalcified and embedded in paraffin. Sections of the embedded specimens (5–6-µm thick) were stained with H&E. The volume of newly-formed bone within each section was analyzed semi-quantitatively with histomorphometric techniques.

### Statistics

All statistical analyses were performed with SPSS13.0 software. Data points were reported as the mean ± standard deviation (SD). The student’s t-test was used to compare two sets of data that were normally distributed, based on normality plots and tests. The Wilcoxon rank-sum test was applied to skewed data distributions. Multiple-variable comparisons were performed with one-way analysis of variance (ANOVA), after the data were checked for equal variance; two-by-two comparisons between the means were performed with the Student Newman-Keuls test. All statistical analyses were performed with at least three biological replicates, unless otherwise stated.

## Results

### ASA pre-treatment inhibited LPS-induced activation of macrophages

**Figure 1:**
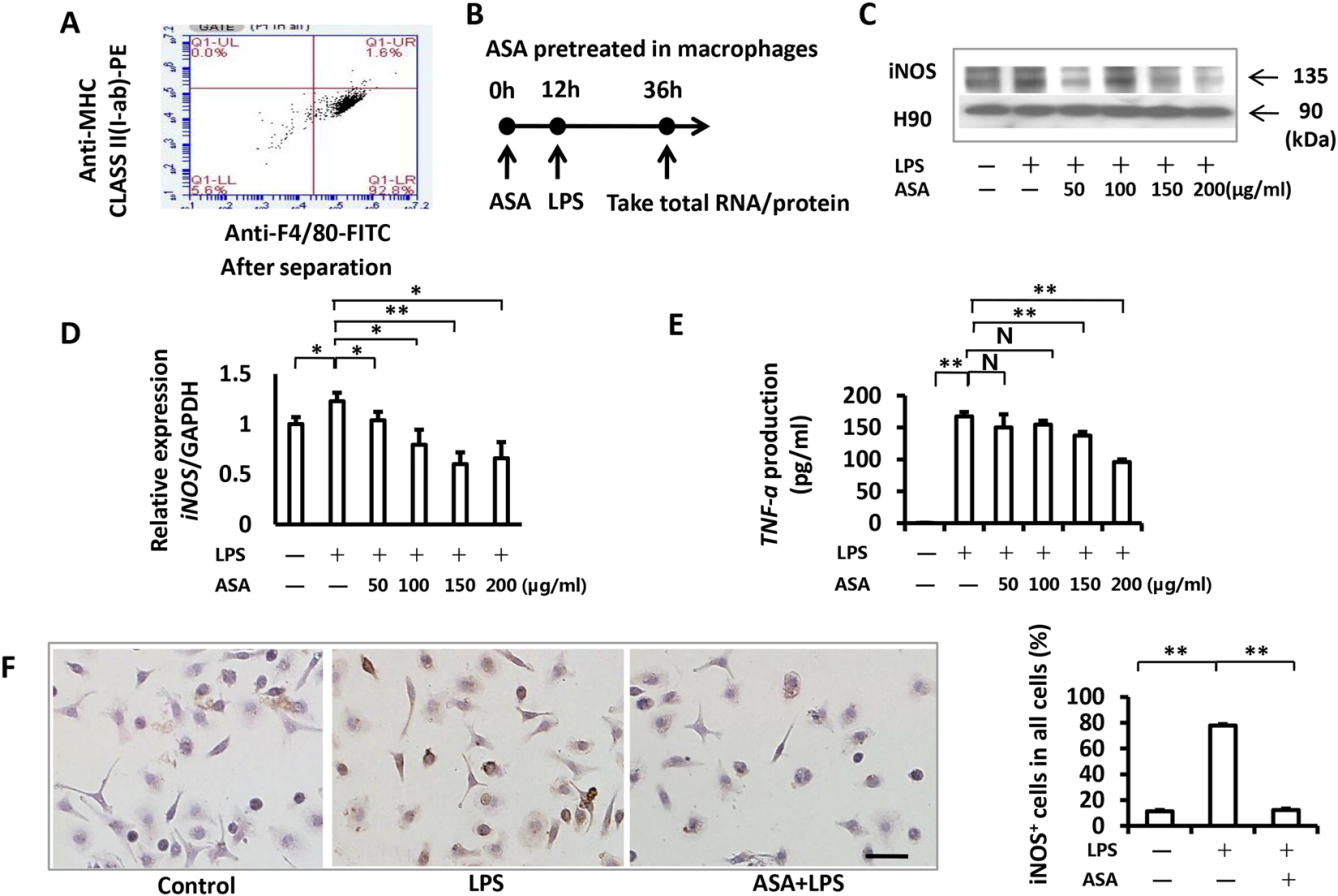
ASA pre-treatment inhibited the activation of LPS-induced in mouse peritoneal macrophage cells. **(A)** The result of flow cytometric analysis showed that >90% of the cells were macrophages after separation. **(B)** Schematic representation showed the timing of ASA and LPS treatment in our experiments. **(C)** The results of western blot showed that before the LPS treatment (1 µg/ml, 24 hrs), the addition of ASA pre-treatment at 200 µg/ml decreased the protein expression of total iNOS. Full-length gels are presented in Supplementary Figure 6. **(D)** The result of PCR showed that 200 µg/ml ASA could significantly decrease the LPS-induced expression of iNOS. We also found ASA below 200 µg/ml concentration could downregulate the expression levels of iNOS induced by LPS. However, 200 µg/ml aspirin downregulated the expression level of iNOS more stablely when compared to the other concentrations. **(E)** The result of ELISA showed the downregulation of TNF-α after ASA pre-treatment (200 µg/ml). **(F)** Immunocytochemical staining assays showed the LPS-induced iNOS positive cells ratio signaficantly increased after LPS inducement (77.79% ± 3.22%) compared to the control group (11.26% ± 1.39%), and 200 µg/ml ASA pre-treatment made the LPS-induced iNOS positive cells ratio decrease (12.29% ± 2.36%). Scale bar = 50 µm. All results are representative of at least three independent experiments. Results were expressed as mean ± standard deviation (SD), and statistical significance was shown as N P > 0.05, *P < 0.05 or **P < 0.01.

To determine a suitable concentration of ASA, we pretreated the isolated peritoneal macrophages cells (Fig. 1A) for 12 hrs with different doses of ASA (50, 100, 150, and 200 µg/ml). These doses were the most commonly used concentration during bone regeneration. Following the ASA treatment, we induced the cells with LPS (Fig. 1B). We found that all these doses of ASA (50, 100, 150, and 200 µg/ml) could significantly decrease the LPS-induced expression of iNOS (Fig. 1C,D; P < 0.05). However, the downregulation effect of iNOS was the most significant and stable in 200 µg/ml aspirin. We also investigated the expression of TNF-α (another product of LPS-induced macrophages) with ELISA analysis. The ELISA results confirmed that 200 µg/ml ASA was the best concentration to inhibited the LPS-induced macrophages (Fig. 1E; P < 0.01). In subsequent assays for this study, we used 200 µg/ml ASA to treat macrophage cells and RAW264.7 cells.

Results from Western blot analyses confirmed that pre-treating with 200 µg/ml ASA could effectively block LPS-induced iNOS expression (Fig. 1C). Immunocytochemical staining showed that the percentage of LPS-induced iNOS positive macrophages significantly increased after LPS stimulation (77.79% ± 3.22%) compared to the control group (11.26% ± 1.39%). However, ASA pre-treatment reduced the percentage of LPS-induced iNOS positive macrophages (12.29% ± 2.36%) (Fig. 1F; P < 0.01). These data suggested that 200 µg/ml ASA inhibited LPS-induced activation of macrophages.

### ASA pre-treatment did not affect expression level of iNOS, ARG1, YM-1,FIZZ in macrophages

We then investigated the expression of ARG1, YM-1 and FIZZ, classic markers of IL-4-induced macrophages. We treated the macrophage cells with different doses of ASA(50, 100, 150, and 200 µg/ml), and the results showed that ASA treatment had no effect on the total RNA and protein expression of FIZZ, YM-1 or ARG1 (Suppl Fig. 2B,C,D,E). In RAW264.7 cells, different doses of ASA (50, 100, 150, and 200 µg/ml) treatment also showed no effect on YM-1, FIZZ expression (Suppl Fig. 3A; P > 0.05). To investigate if ASA treatment would influence macrophages without LPS inducement, we treated cells with 200 µg/ml ASA for 36 hrs. The results of flow cytometric analysis showed that ASA at 200 µg/ml had no effects on the apoptosis in RAW264.7 cells (Suppl Fig. 4A, P > 0.05). Moreover, Western blot and PCR also showed that 200 µg/ml ASA treatment had no effect on the expression of iNOS, YM-1, FIZZ or TNF-α in macrophage cells.

### ASA inhibited LPS-induced macrophage via the COX_2_/PGE_2_/EP_2_/NF-κB cascade

**Figure 2:**
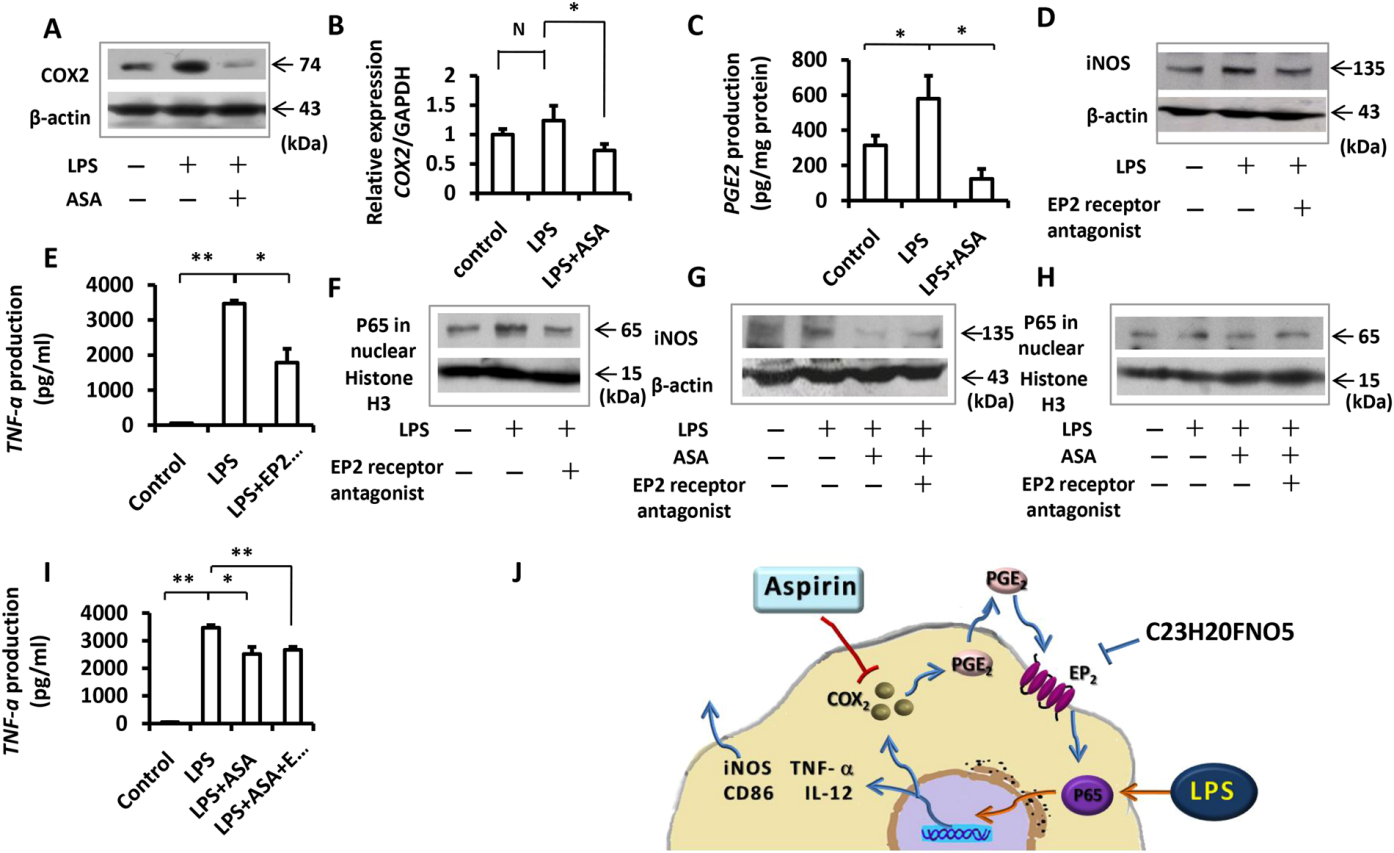
COX_2_-PGE_2_-EP_2_-NF-κB pathway played a pivotal role in the activation of LPS-induced RAW264.7 cells. **(A)** LPS treatment increased the protein expression of COX_2_, whereas ASA pre-treatment decreased the protein expression of COX_2_. Full-length gels are presented in Supplementary Figure 7. **(B)** The PCR result also showed the upgregulation of COX_2_ after LPS treatment and the downgregulation of COX_2_ in the presence of ASA pre-treatment. **(C)** The ELISA result showed LPS treatment promoted the expression level of PGE_2_ while ASA pre-treatment decreased the expression level of PGE_2_. **(D)** The upregulation of iNOS after LPS treatment could be inhibited by blocking the EP_2_ receptor with EP_2_ antagonist. Full-length gels are presented in Supplementary Figure 7. **(E)** The result of ELISA showed EP_2_ antagonist decreased the expression level of TNF-α induced by LPS. **(F)** In the presence of LPS, EP_2_ antagonist blocked P65 nuclear translocation. Full-length gels are presented in Supplementary Figure 7. **(G)** The result of western blot showed the downregulation of iNOS after ASA + LPS treatment could not be totally blocked by EP_2_ antagonist. Full-length gels are presented in Supplementary Figure 7. **(H)** EP_2_ antagonist also could not totally block P65 nuclear translocation during ASA + LPS treatment. Full-length gels are presented in Supplementary Figure 7. **(I)** The result of ELISA confirmed that EP_2_ antagonist could not totally block the downregulation of TNF-α production of LPS-induced macrophages by ASA pre-treatment. **(J)** All results above indicated that the COX_2_-PGE_2_-EP_2_-NF-κB pathway indeed involved in the process of LPS-induced macrophages activation. Therefore ASA could inhibit the activation of LPS-induced macrophages by decreasing the expression of COX_2_ as showed in this schematic representation. However, the results also showed that ASA could make this effect via more than one pathway. All results are representative of at least three independent experiments. Results were expressed as mean ± standard deviation (SD), and statistical significance was shown as *P < 0.05 or **P < 0.01.

Next, we explored the mechanisms underlying ASA inhibition of LPS-induced macrophage activation in RAW264.7 cells. Because ASA was a well-known COX_2_ inhibitor, we first sought a relationship between COX_2_ and the NF-κB pathway. We found that, as one of the products of the NF-κB pathway, COX_2_ could enhance the expression of NF-κB *via* a COX_2_/PGE_2_/EP_2_/NF-κB positive feedback loop^27^. We found that ASA pre-treatment reduced the LPS induction of COX_2_, as indicated from Western blot (Fig. 2A) and quantitative real time PCR analyses (Fig. 2B; P < 0.05). As a downstream product of COX_2_, PGE_2_ expression also decreased with ASA pre-treatment (Fig. 2C; P < 0.05). Next, we investigated whether this feedback loop was involved in the LPS-induced activation of macrophages. We blocked the feedback loop with an EP_2_ antagonist before inducing the cells with LPS. The results showed that LPS-induced upregulation of iNOS could be inhibited by blocking EP_2_ receptor (Fig. 2D). Moreover, the EP_2_ antagonist also blocked P65 nuclear translocation (Fig. 2F). To make sure the stabilization of the result that COX_2_/PGE_2_/EP_2_/NF-κB pathway was involved, we investigated the expression of TNF-α with an ELISA. The results showed that the EP_2_ antagonist also reduced the TNF-α levels (Fig. 2E; P < 0.05). These data suggested that the COX_2_/PGE_2_/EP_2_/NF-κB pathway was involved in the LPS-induced activation of macrophages. Thus, ASA inhibited LPS-induced macrophage by downregulating the expression of COX_2_ (Fig. 2J). However, we found that the downregulation of both iNOS expression and P65 nuclear translocation caused by ASA could not be completely reproduced with the EP_2_ antagonist (Fig. 2G–I; P < 0.05). These results implied that ASA inhibited LPS-induced macrophage activation through more than one pathway.

### ASA inhibited LPS-induced macrophage via directly inhibiting IκK/IκB/NF-κB pathway

**Figure 3:**
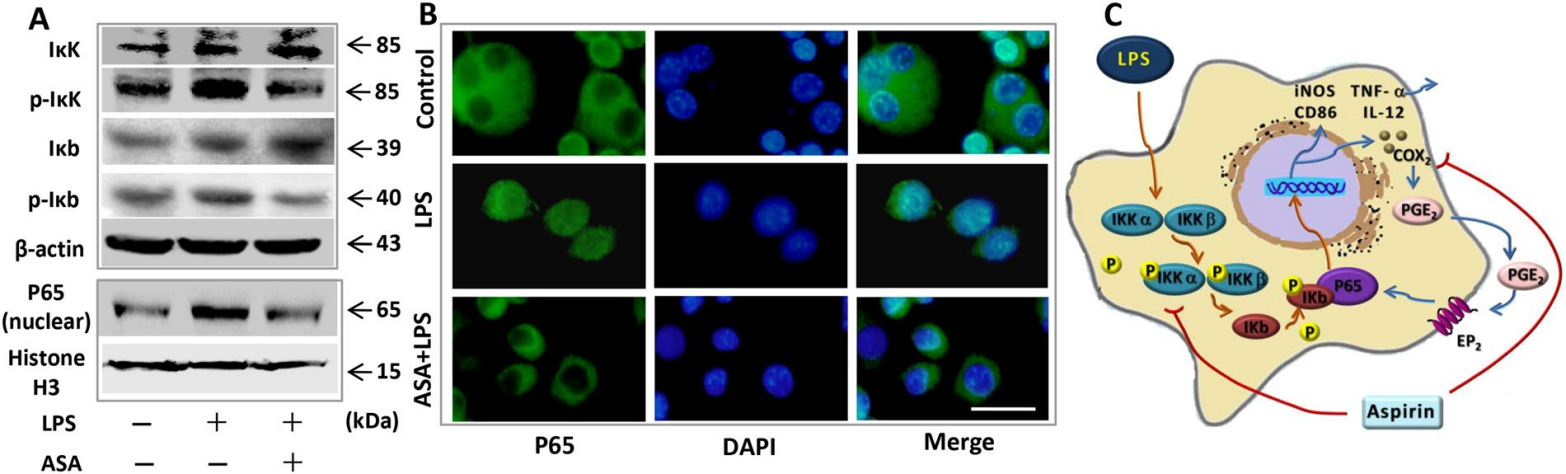
ASA at 200 µg/ml inhibited the activation of LPS-induced macrophages via NF-κB pathway. **(A)** LPS treatment increased the protein expression of p-IκK and p-Iκb. In contrast ASA pre-treatment inhibited the upregulation of p-IκK and p-Iκb induced by LPS. Full-length gels are presented in Supplementary Figure 8. **(B)** For the immunofluorescence assay. From left to right represent the P65 protein, nucleus and the merged images with P65 protein and the nucleus. Results showed LPS promoted P65 nuclear translocation whereas ASA pre-treatment inhibited this process. Scale bar = 20 µm. **(C)** Based on all results as described above, ASA inhibited the activation of LPS-induced macrophages by both COX_2_-PGE_2_-EP_2_-NF-κB pathway and NF-κB pathway. All results are representative of at least three independent experiments.

Next, we investigated another pathway that might have contributed to the ASA inhibition of LPS-induced macrophage activation. Previous studies reported that ASA could inhibit the activity of IκB kinase(IκK)-β^28, 29^, the key kinases in the NF-κB pathway. Therefore, we investigated the expression levels of IκK, p-IκK, IκB, and p-IκB proteins. We found that LPS treatment increased the expression of p-IκK and p-IκB proteins, and that ASA inhibited this upregulation (Fig. 3A). We also found that LPS promoted P65 nuclear translocation, and this effect was inhibited with ASA treatment (Fig. 3A,B).

Taken together, these data indicated that both the IκK/IκB/NF-κB pathway and the COX_2_/PGE_2_/EP_2_/NF-κB positive feedback loop were involved in the ASA inhibition of LPS-induced macrophage activation (Fig. 3C).

### ASA treatment improved bone regeneration in the rat mandibular defect model

**Figure 4:**
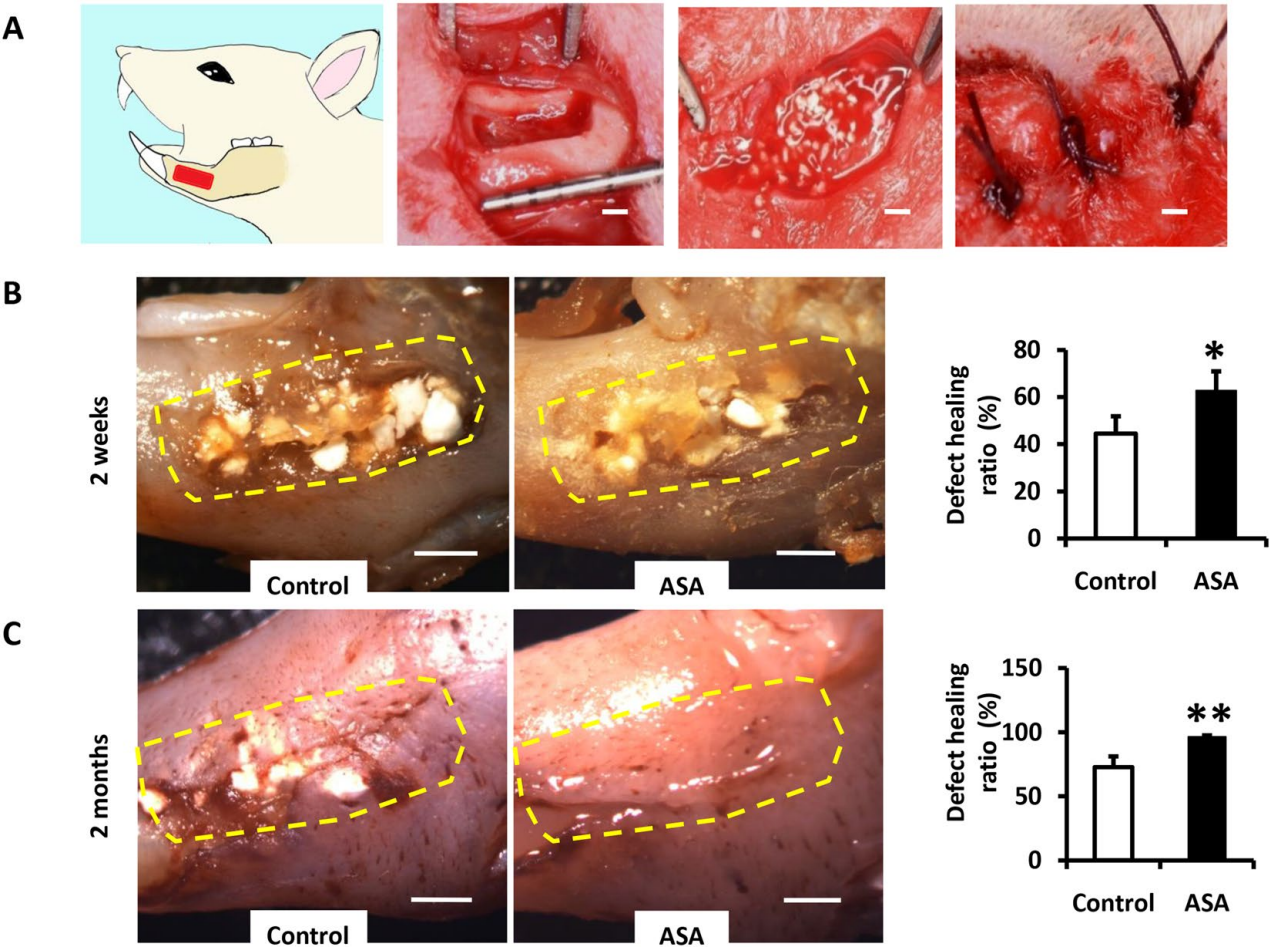
ASA at 200 µg/ml showed favorable therapeutic effects in promoting alveolar bone defect healing. **(A)** Schematic representation showed the process of establishment of alveolar bone defect model in a rat. Scale bar = 1 mm. **(B)** Two weeks after the surgery, the healing speed of defects in ASA group were much faster compared to the control group. Scale bar = 1 mm. **(C)** Two months after the surgery, ASA group showed a great advantage in defect healing ratio compared to the control group. Scale bar = 1 mm. The dotted boxes in **(B)** and **(C)** are the defect area we made in the surgery at the beginning. All results are representative of at least three independent experiments. Results were expressed as mean ± standard deviation (SD), and statistical significance was shown as *P < 0.05 or **P < 0.01.

We then investigated the immunomodulatory functions of ASA-pretreated macrophages *in vivo* in a rat mandibular defect model. First, we established a 5 mm × 2 mm × 1 mm defect in the rat alveolar bone, between the incisors and molars. Then, we used hydrogel as a carrier for administering 200 µg/ml aspirin. This mixture was used to fill the defects in the aspirin group; the hydrogel mixed with DMSO was used in control groups. We covered the mixture with HA/TCP in both groups as a scaffold. Finally, we closed the skin incisions with 4-0 absorbable sutures (Fig. 4A). Two weeks after surgery, we found that healing occurred much more rapidly in the aspirin group than in the control group (Fig. 4B; P < 0.05). In addition, two months after surgery, aspirin administration provided a great advantage by increasing the rate of defect healing compared to the control group (Fig. 4C; P < 0.01). These findings showed that aspirin treatment benefited bone formation in the repair of bone defects.

### ASA inhibited LPS-induced iNOS positive macrophage in the rat mandibular bone defect model

**Figure 5:**
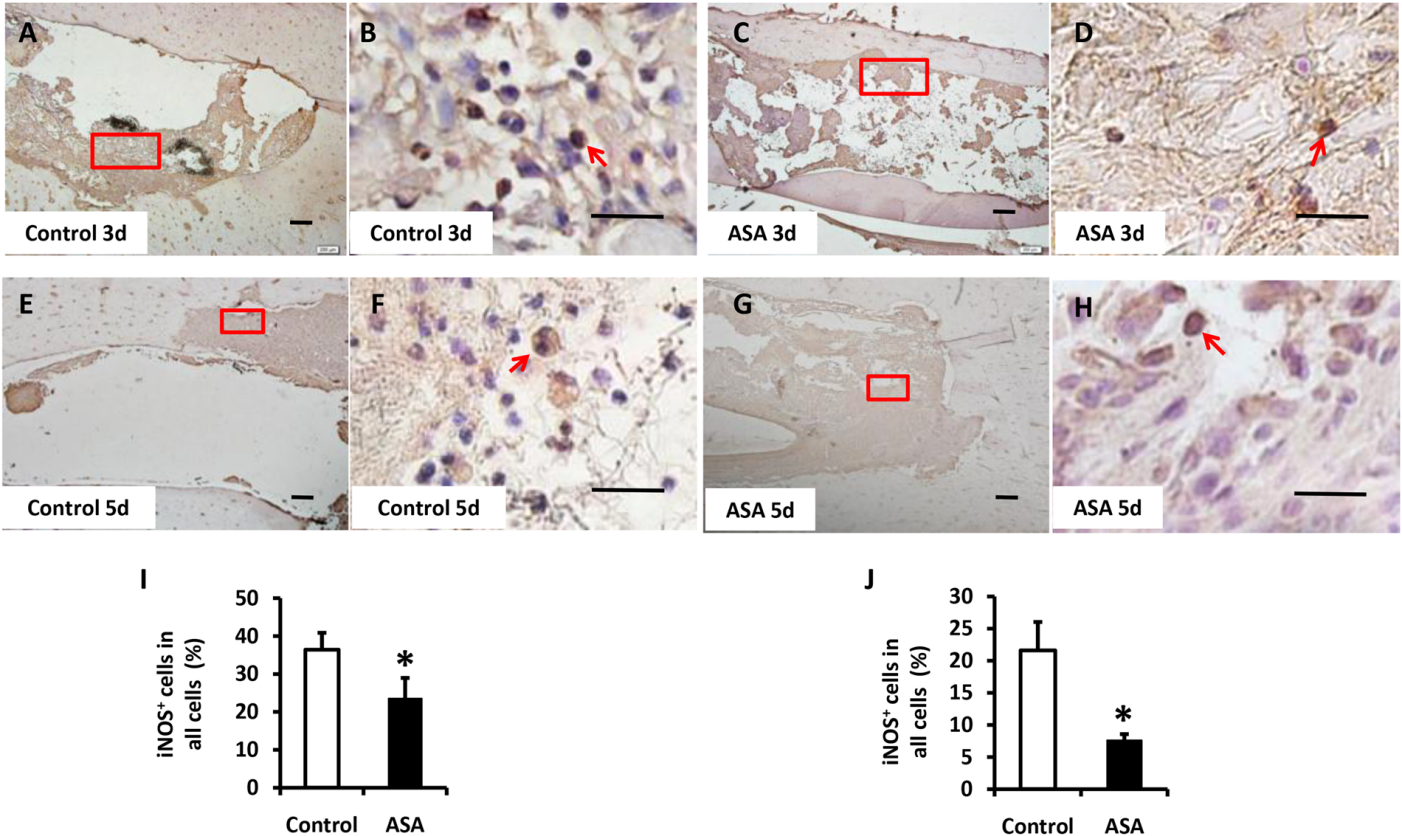
ASA inhibited LPS-induced iNOS^+^ macrophages during the early inflammation *in vivo*. **(A–D,I)** Immunocytochemical staining assays showed by the third day after the surgery, both control group and ASA group showed an overexpression of iNOS. However the iNOS^+^ cells ratio was lower in ASA group compared to the control group. Scale bar = 200 µm in **(A,C)** and scale bar = 20 µm in **(B,D)**. **(E–H,J)** By the fifth day after surgery, the iNOS^+^ cells ratio decreased in both group whereas ASA group still showed a lower iNOS^+^ cells ratio than control group. Scale bar = 200 µm in **(E,G)** and scale bar = 20 µm in **(F,H)**. The *red boxes* in **(A)** and **(B)** are representative of the magnified fields as shown in the *lower panel*. All results are representative of at least three independent experiments. Results were expressed as mean ± standard deviation (SD), and statistical significance was shown as *P < 0.05 or **P < 0.01.

We next examined whether aspirin treatment could reduce the number of local LPS-induced iNOS positive macrophages *in vivo*. On the 3^rd^ day after surgery, the percentage of LPS-induced iNOS positive macrophages increased in both groups (Fig. 5A–D). However, compared to the control group, the aspirin group had a lower percentage of iNOS-expressing cells (Fig. 5I; P < 0.05). By the 5^th^ day after surgery, the percentage of LPS-induced iNOS positive macrophages had decreased with time passed by (Fig. 5E–H). The percentage of iNOS-expressing cells remained lower in the aspirin group than in the control group (Fig. 5J; P < 0.05).

### ASA increased newly regenerated bone volume in the rat mandibular bone defect model

**Figure 6:**
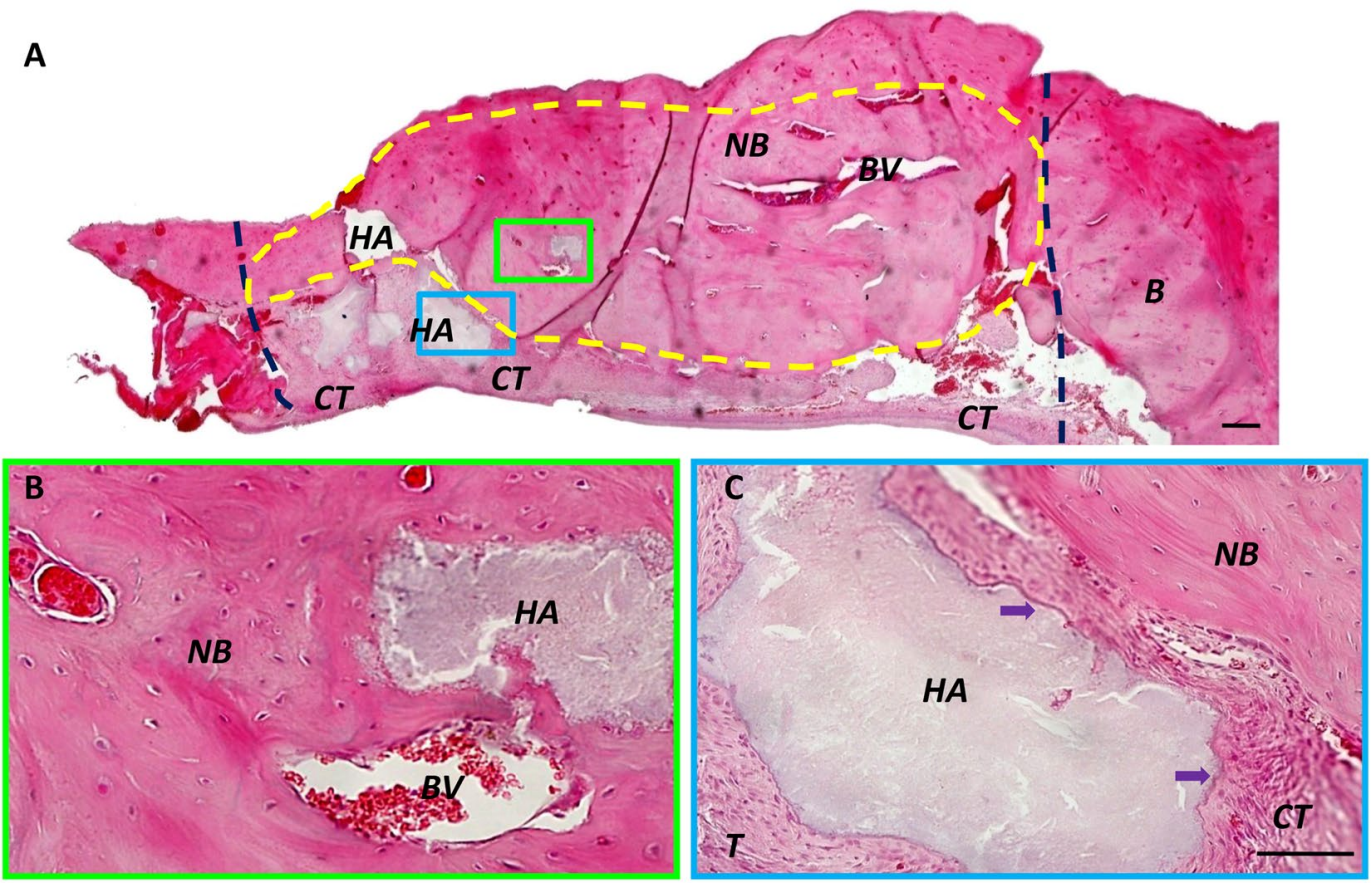
HA/TCP mediated moderate new bone formation in mandibular bone defects. **(A)** Two months after surgery, the control group treated with HA/TCP alone showed moderate amounts of new bone in the defects. HA/TCP was also detected in the mineralized tissues (dark blue lines show the margins of the bone defect made in surgery). **(B)** Higher magnification of the area enclosed in a green rectangle in (**A**) shows newly formed bone was detected around HA/TCP particles. There were abundant blood vessels in the newly formed bone. **(C)** Control HA/TCP specimens show large volumes of HA/TCP particles remaining in the bone defect area, around connective tissues (purple arrows; the area enclosed with a blue rectangle in (**A**). HA = HA/TCP particles, NB = new bone, CT = connective tissue, BV = blood vessel. Scale bar = 200 µm in **(A)** and Scale bar = 50 µm in **(B,C)**.

**Figure 7:**
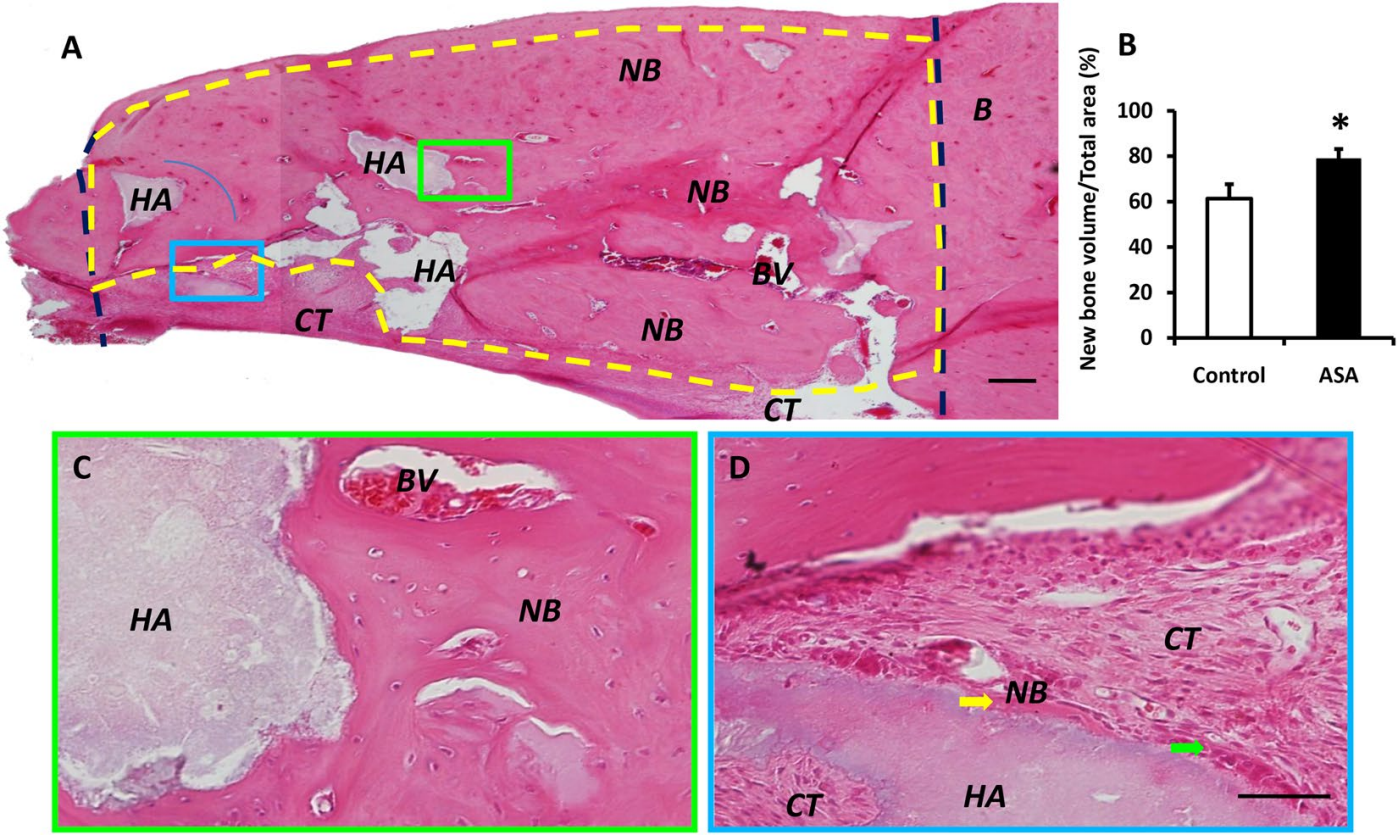
Aspirin increased newly regenerated bone in mandibular bone defects. **(A)** The HA/TCP + aspirin group displayed nearly full restoration of the defect (dark blue lines show the margins of the bone defect made in surgery). **(B)** Aspirin treatment increased the new bone volume compared to the HA/TCP control group. **(C)** Higher magnification of the area enclosed in a green rectangle in (**A**) shows a large volume of newly formed bone and blood vessels. **(D)** The area enclosed in a blue rectangle in (**A**) shows that active bone formation continued (yellow arrows), and osteoblasts are observed around the surfaces of HA/TCP particles (green arrows). HA = HA/TCP material, NB = new bone, CT = connective tissue, BV = blood vessel. Scale bar = 200 µm in **(A)**; scale bar = 50 µm in **(C,D)**. The results are representative of at least three independent experiments. Results are expressed as the mean ± standard deviation (SD); *P < 0.05.

For long-term observations, mandibular bone specimens were prepared, and H&E stained sections were assayed at two months after surgery. Images were captured at a lower magnification to examine the entire defect area (Figs 6 and 7). The control group with HA/TCP-treatment alone exhibited new bone formation (Fig. 6A). At higher magnification, despite the moderate amount of new bone formation in the control group (Fig. 6B), a large number of HA/TCP particles remained among the connective tissues (Fig. 6C). In contrast, the aspirin group displayed nearly full restorations of the defect (Fig. 7A), and the newly-formed bone was significantly improved compared to that observed in the control group. A semi-quantitative analysis showed that a significantly higher percentage of new bone formation occurred in the regenerated defect site in the aspirin group (78.73% ± 4.43%) compared to the control group (61.31% ± 6.36%) (Fig. 7B; P < 0.05). At higher magnification, a large volume of newly formed bone and blood vessels was observed (Fig. 7C). We also observed osteoblasts around the surfaces of HA/TCP particles (green arrows, Fig. 7D), which indicated that active bone formation had continued (yellow arrows, Fig. 7D).

## Discussion

As a classic NSAID, aspirin has been widely used for clinical applications to reduce inflammation, relieve pain, prevent heart attacks, and prevent blood clot formation. Aspirin’s mechanism of action is to inhibit COX, which is the rate-limiting enzymatic step in the conversion of arachidonic acid to pro-inflammatory prostaglandins. Based on its involvement in multiple biological pathways, over the last decades, studies have focused on the roles of aspirin in the process of bone metabolism.

Currently, the effects of NSAID on bone healing remain highly debated. Epidemiologic studies have revealed that, to date, individuals that had regularly used aspirin had significantly higher bone mineral density compared to non-users, based on quantitative computed tomography data^30^. Moreover, a recent study highlighted the positive effect of aspirin in the treatment of ovariectomy-induced osteoporosis. Aspirin was shown to activate osteoblasts by increasing telomerase activity, and simultaneously, it inhibited osteoclasts^31^. On the other hand, many animal studies have shown that traditional NSAIDS (nonspecific) such as aspirin, indomethacin, and ibuprofen impeded bone healing^32^. Those controversial results may be due to differences in the specific drug used, the drug preparations, the modes of administration, and the degrees of COX_2_ inhibition. Recently, Wada K *et al*. used a unique local drug delivery system (SA-PAEs) that released ASA in a localized, controlled, sustained manner. SA-PAEs locally resolved inflammation and increased bone formation *in vivo* in diabetic and normoglycemic animals^33^. In the present study, we aimed to confirm that locally delivered aspirin was a suitable method for promoting bone reparation and regeneration.

Our previous studies lent support to the notion that aspirin treatment could promote osteogenesis, both *in vitro* and *in vivo*. These benefits were attributed to the involvement of aspirin in multiple biological pathways, including its inhibition of COX_2_ and PGE_2_, its impact on osteoclasts activity, and its reduction of the abundances of local inflammatory factors and T cells. However, the influence of aspirin on macrophages during inflammation remained unknown.

Macrophages, first described by Metchnikoff over 100 years ago, comprise a major component of the immune cell population. Macrophages are professional phagocytes that are highly specialized in removing dying or dead cells and cellular debris. They play a critical role in innate immunity, and they contribute to the initiation of adaptive immunity by recruiting other immune cells^34^. Recent studies have confirmed that macrophages were involved in anti-inflammatory processes and bone remodeling^35, 36^. In this study, we found that ASA treatment inhibited macrophage polarization to the LPS-induced macrophages. It is well known that LPS can provoke macrophages to differentiate into the inflammatory phenotype *via* the NF-κB pathway. We found that, when the NF-κB pathway was activated, COX_2_ secretion increased. As a result, PGE_2_ was upregulated, and its binding to the EP_2_ receptor promoted the activation of nuclear p65 (NF-κB)^27^. This led to the NF-κB/COX_2_/PGE_2_/EP_2_/NF-κB positive feed-back loop, which increased the number of LPS-induced macrophages. We confirmed the existence of the positive feed-back loop with an EP_2_ antagonist. As a classic COX_2_ inhibitor, ASA can inhibit LPS-induced macrophages by the downregulation of COX_2_. When we added the EP_2_ receptor antagonist, it could not completely reproduce the aspirin-mediated reduction of LPS-induced macrophages. These data suggested that another pathway was involved in the process. We then demonstrated that aspirin could also inhibit the NF-κB pathway directly, by inhibiting the activity of IκK-β^28, 29^. Collectively, these results suggested that aspirin inhibited the activation of macrophages induced by LPS *via* both the IκK/IκB/NF-κB pathway and the COX_2_/PGE_2_/EP_2_/NF-κB feedback loop. Moreover, we confirmed that iNOS expression was inhibited by pre-treating with ASA.

## Conclusion

In summary, the present study showed that ASA treatment significantly promoted the healing of rat alveolar bone defects by reducing the abundance of LPS-induced macrophages in the early stages of inflammation, without affecting the expression of ARG1/FIZZ/YM-1 in macrophage. ASA inhibited the activation of LPS-induced macrophages *via* the IκK/IκB/NF-κB pathway and the COX_2_/PGE_2_/EP_2_/NF-κB positive feedback loop. These findings suggested that ASA treatment might be a promising therapeutic strategy for treating inflammation and promoting bone remodeling and repair in future clinical applications. However, it has become increasingly clear that COX_2_ function is critical for bone regeneration. Therefore, future research is needed to define the role of COX_2_ in bone regeneration and the feasibility and efficiency of administering ASA in the clinic.

## Electronic supplementary material

Supplementary information
