# Supplementary material for: Aspirin inhibits LPS-induced macrophage activation via the NF-κB pathway
Source: Sci Rep. 2017 Sep 14;7:11549. doi: 10.1038/s41598-017-10720-4 (PMC5599518; doi:10.1038/s41598-017-10720-4)
Supplement: Supplementary file 1 — Supplementary information [file 41598_2017_10720_MOESM1_ESM.pdf]

Supporting Online Material for

Aspirin inhibits LPS-induced macrophage activation *via* the NF-κB pathway

Yitong Liu[1], Silian Fang[2], Xiaoyan Li[1], Jie Feng[1], Juan Du[1], Lijia Guo[2], Yingying Su[3], Jian Zhou[1], Gang Ding[4], Yuxing Bai[5], Songling Wang[6], Hao Wang[3], Yi Liu[1#]

[#]To whom Correspondence should be addressed Email: lililiuyi@163.com

Supplementary Methods

Chemicals and antibodies. Aspirin (over 99% purity) and dimethyl sulfoxide (DMSO) were purchased from Sigma-Aldrich (St. Louis, MO, USA). Ultrapure lipopolysaccharide *from Porphyromonas gingivalis* (LPS) was purchased from InvivoGen (San Diego, CA, USA). Antibodies specific for iNOS, p65 NF-κB, IκK, IκB, phosphorylated IκK (p-IκK), p-IκB and p-p65 were purchased from Cell Signaling Technology (Boston, MA, USA). The anti-FIZZ antibody, anti-ARG1 antibody, anti-YM-1 antibody were purchased from Abcam (Cambridge, Cambs, UK). An anti-β-actin antibody , anti-Heat shock protein90 (H90) antibody were purchased from Sigma-Aldrich (St. Louis, MO, USA).

Kits. The Mouse Peritoneal Macrophage Isolation Kit was purchased from Miltenyi Biotec (Bergisch Gladbach, Germany). The Annexin V-PI Apoptosis Detection Kit was purchased from BD Bioscience (Franklin Lakes, NJ, USA). The Total Nitric Oxide and Nitrate/Nitrite Parameter Assay Kit was purchased from CUSABIO (Wuhan, China).

Isolation of mouse peritoneal macrophages. Six-to-eight-week-old female C57BL/6 mice were given an injection of 2 ml thioglycollate medium (4%), Five days after injection we collected all the cells obtained by peritoneal lavage. Using the Macrophage Isolation Kit (Peritoneum), macrophages were isolated by depletion of non-targget cells. The magnetically labeled non-target cells were depleted by retaining them within a MACS Column in the magnetic field of a MACS Separator, while the unlabeled macrophages were passed through the column. The separated macrophages were determined by flow cytometric analysis using anti-F4/80-FITC antibody and anti-MHC ClassII(I-ab)-PE antibody, which suggested that >90% of the cells were macrophages. The macrophage cells were cultured in the presence of RPMI medium (Invitrogen, Carlsbad, CA) supplemented with 15% heat-inactiated fetal bovine serum (FBS; Equitech-Bio, Kerrville, TX), 5% pen/Strep (Biofluids, Inc.) and 5% glutamine (Invitrogen). Cultures were incubated at 37℃, with 5% CO_2, in a humidified atmosphere. The cell medium was changed every 2-3 days. All cells were induced within one week after isolation.

RAW264.7 cells culture. RAW264.7 cells (China Infrastructure of Cell Line Resources, Beijing, China) were cultured in Dulbecco’s modified Eagle’s medium (Invitrogen, Carlsbad, CA，USA) with 10% fetal bovine serum (FBS; Equitech-Bio, Kerrville, TX), 2 mM L-glutamine (Invitrogen), 100 U/ml penicillin and 100 μg/ml streptomycin (Biofluids, Inc.). Cultures were incubated at 37 ℃, with 5% CO_2, in a humidified atmosphere. The cell medium was changed every 2-3 days. Cells were passaged when they became 70% to 80% confluent.

Induction of macrophage cells by LPS . We treated both mouse peritoneal macrophages and RAW264.7 cells with 1 μg/ml LPS for 24 hrs, and the iNOS, ARG1, YM-1, FIZZ expression levels were determined with quantitative real time PCR, Western blot, and the iNOS expression levels were also determined with ELISA analyses. The ratios of iNOS positive cell number in total RAW264.7 cells were calculated.

For NF-κB pathway time course we treated RAW264.7 cells with 1 μg/ml LPS for 3 hrs, 6 hrs, 12 hrs, 24 hrs and 48 hrs, and the p-IκK, p-IκB and p-p65 expression levels were determined with Western blot.

ASA pre-treatment inhibited LPS-induced activation of RAW264.7. RAW264.7 cells were seeded at a density of 2×10^5 cells/well in a 6-well plate, and cultured in a 37 ℃ in a 5% CO_2 incubator. After treating with ASA (0, 50, 100, 150, or 200 μg/ml) for 12 hrs, the cells were induced to become LPS-induced macrophages by adding 1 μg/ml LPS, and incubating for another 24 hrs. The negative control was RAW264.7 cells challenged with phosphate buffered saline (PBS) and DMSO. The iNOS expression levels were determined with quantitative real time PCR, Western blot and ELISA analyses. Moreover, the FIZZ and YM-1 expression levels were determined with quantitative real time PCR.

ASA treatment without LPS inducement. Macrophage cells were seeded at a density of 2×10^5 cells/well in a 6-well plate after isolation, RAW264.7 cells were seeded at a density of 2×10^5 cells/well in a 6-well plate, and then cultured in a 37 ℃ in a 5% CO_2 incubator. Then we treated the cells with 200 μg/ml ASA for 36 hrs. The negative control was macrophages cells and RAW264.7 cells challenged with DMSO. The

iNOS, YM-1, FIZZ expression levels were determined with quantitative real time PCR, Western blot and the TNF-α expression levels were determined with quantitative real time PCR. And the fraction of apoptotic RAW264.7 cells was detected with flow cytometry. Briefly, cells were resuspended in 100 μl binding buffer with 5 μl Annexin V-FITC and 5 μl propidium iodide (PI) solution (BD Bioscience), then incubated for 15 min in the dark. Cell preparations were immediately analyzed with flow cytometry (FACSCalibur, BD Bioscience). The experiments were performed at 4 ℃ in the dark.

Quantitative real-time PCR analysis. After the cell treatments, total RNA was extracted from each group with Trizol reagent (Invitrogen, Carlsbad, CA, USA). We synthesized cDNA from 2 μg aliquots of total RNA, oligo(dT) (Invitrogen, Carlsbad, CA, USA), and RNaseOUTTM Recombinant Ribonuclease Inhibitor (Invitrogen, Carlsbad, CA, USA), according to the manufacturer's protocol. Real-time PCR reactions were performed with the Power SYBR® Green PCR Master Mix (Life Technologies, Warrington, UK) and primers that targeted iNOS, TNF-α, YM-1, FIZZ and ARG1 (Suppl Table 1.).

Western blot analysis. Total protein was extracted with NE-PER nuclear and cytoplasmic extraction reagents (Thermo). Nuclear proteins were isolated with a mammalian nuclear and cytoplasmic protein extraction kit (TransGen Biotech). Next, 50-100 μg aliquots of protein were separated on 10% polyacrylamide-SDS gels (Pplygen) and transferred to Immobilon™-P membranes (Millipore). After blocking with TBS/5% nonfat dry milk (Pplygen) for 1 h, the membrane was incubated with antibodies against mouse PPARγ (Cell Signaling Technology), histone H3 (Bioworld), and β-actin (Sigma) overnight at 4 ℃. Next, membranes were incubated with horseradish peroxidase-conjugated secondary antibodies (Pierce, Malibu, CA, USA) for 1 h at room temperature. Antibody binding was visualized with an enhanced chemiluminescence kit, according to the manufacturer's protocols (Pierce).

ELISA analysis. Cell culture supernatants were collected to detect the expression of iNOS with corresponding ELISA kits. The assays were performed according to the manufacturer's instructions.

Immunohistochemistry. RAW264.7 cells (2×10^5 cells/well) were seeded into 12-well plates that contained glass coverslips. Control groups were pretreated with DMSO, and the ASA groups were pretreated with 200 μg/ml ASA for 12 hrs. Then, 1 μg/ml LPS was added for another 24 hrs. Next, the glass coverslips were harvested, fixed in 4% paraformaldehyde (PFA). To reduce non-specific staining, all sections were incubated with 3% hydrogen peroxide for 10 min at room temperature and blocked with 10% serum for 60 min at 37℃. Then, the pretreated sections were incubated with primary antibodies (50 μg/ml) at 4℃ overnight. Biotinylated secondary antibodies (1 μg/ml) were added, and specimens were incubated at room temperature for 1 h. Finally, the horseradish peroxidase complex was added with the diaminobenzidine substrate for visualization. After cells were counterstained, slides were observed under a light microscope (OLYMPUS, Tokyo, Japan). Semi-quantification was performed by counting the number of positively stained cells in at least five random fields.

Statistics. All statistical analyses were performed with SPSS13.0 software. Data points were reported as the mean±standard deviation (SD). The student's t-test was used to compare two sets of data that were normally distributed, based on normality plots and tests. The Wilcoxon rank-sum test was applied to skewed data distributions. Multiple-variable comparisons were performed with one-way analysis of variance (ANOVA), after the data were checked for equal variance; two-by-two comparisons between the means were performed with the Student Newman-Keuls test. All statistical analyses were performed with at least three biological replicates, unless otherwise stated.

Supplementary figure.1

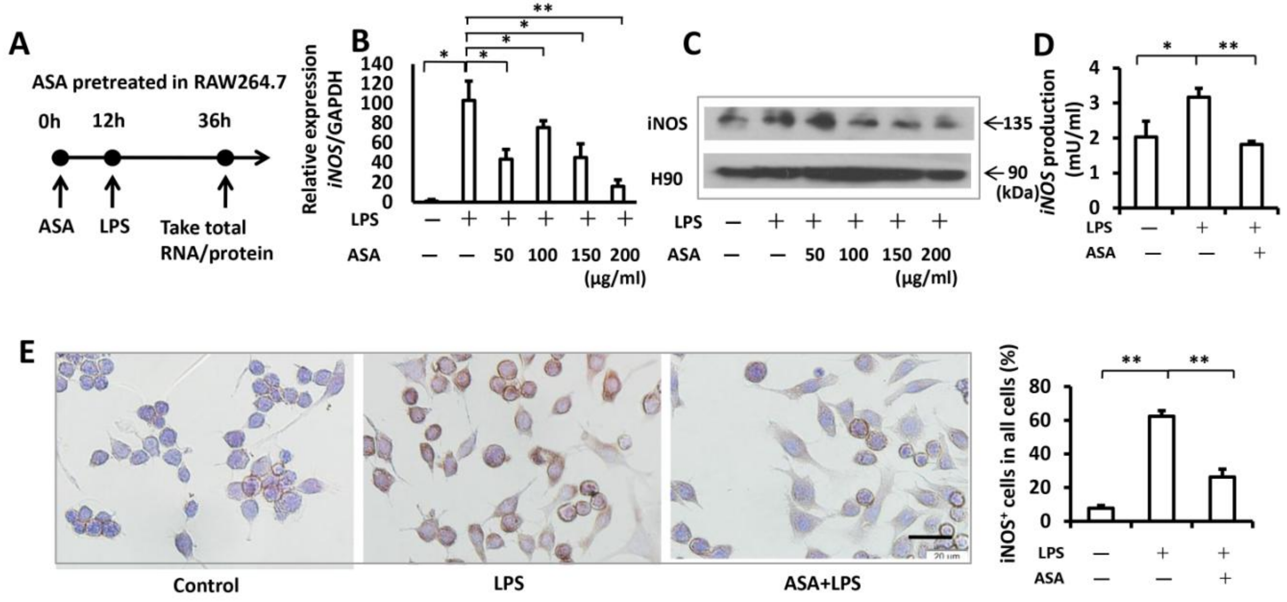

Supplementary Figure. 1. ASA pre-treatment inhibited the activation of LPS-induced macrophages in RAW264.7 cells.
(A) Schematic representation shows the timing of ASA and LPS treatment in our experiments. **(B)** Before the LPS treatment (1µg/ml, 24 hours), the addition of ASA pre-treatment (50-200µg/ml, 12 hours) significantly decreased the total RNA expression of iNOS in RAW264.7 and ASA at 200µg/ml decreased RNA expression of iNOS most. **(C)** The results of western blot showed that ASA at 200µg/ml decreased the protein expression of total iNOS. Full-length gels are presented in Supplementary Figure 9. **(D)** The downregulation of iNOS after 200µg/ml ASA pre-treatment could be confirmed by ELISA result.**(E)** Immunocytochemical staining assays showed the LPS-induced iNOS positive cells ratio signaficantly increased after LPS inducement (62.32%±3.36%) compared to the control group (7.80%±1.52%), and 200µg/ml ASA pre-treatment made the LPS-induced iNOS positive cells ratio decrease (26.34%±4.60%). Scale bar=20 µm.
All results are representative of at least three independent experiments. Results were expressed as mean±standard deviation (SD), and statistical significance was shown as * $P<0.05$ or ** $P<0.01$.

Supplementary figure.2

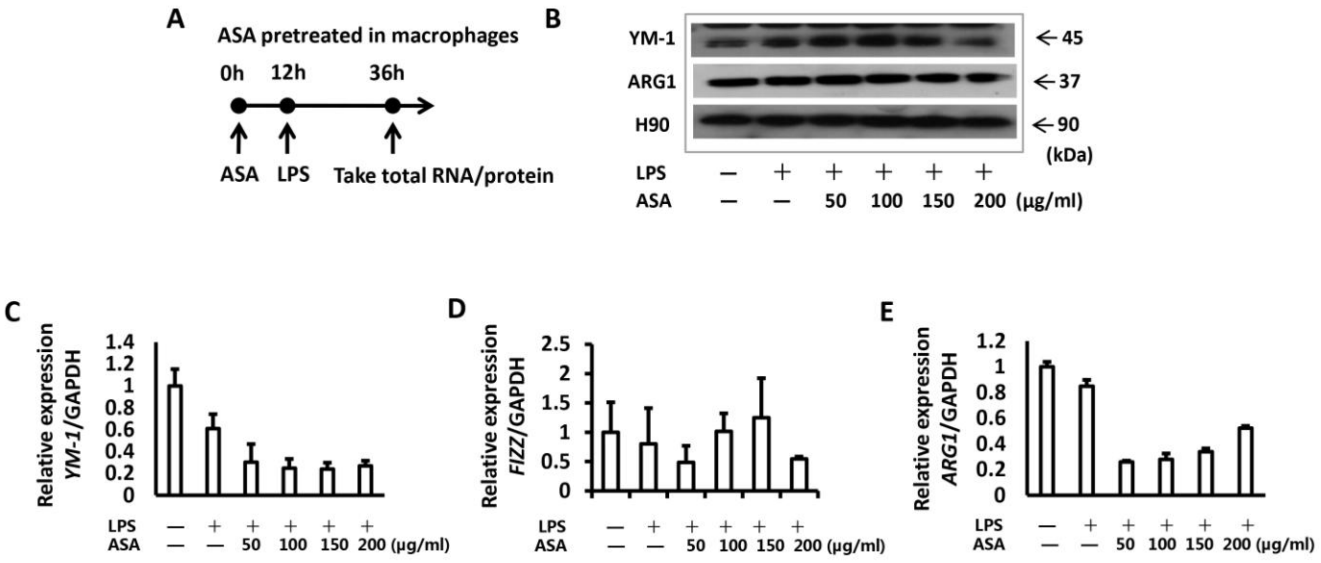

Supplementary Figure. 2. ASA pre-treatment had no effect on expression of FIZZ, YM-1 and ARG1 in macrophages when induced by LPS.
(A) Schematic representation shows the timing of ASA and LPS treatment in our experiments. **(B)** The results of western blot showed that ASA pre-treatment had no effect on protein expression of YM-1 and ARG1. Full-length gels are presented in Supplementary Figure 10. **(C,D,E)** The results of PCR showed that ASA pretreatment did not affect the total RNA expression of FIZZ, YM-1 and ARG1.
All results are representative of at least three independent experiments. Results were expressed as mean ± standard deviation (SD).

Supplementary figure.3

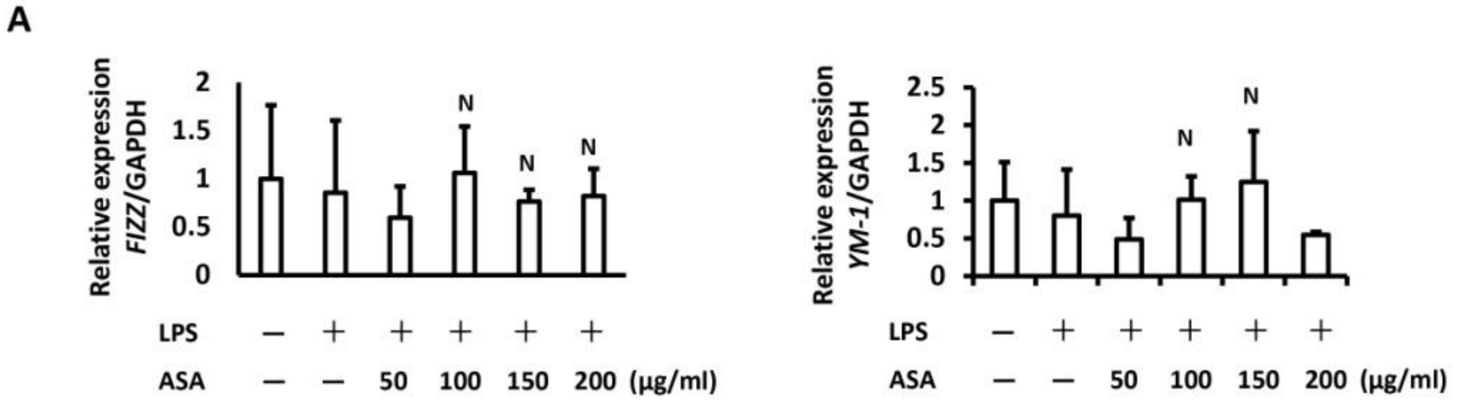

Supplementary Figure. 3. ASA pre-treatment had no effect on expression of FIZZ, YM-1 in RAW264.7 when induced by LPS.
(A) The results of PCR showed that ASA pretreatment did not affect the total RNA expression of FIZZ, YM-1 in RAW264.7.
All results are representative of at least three independent experiments. Results were expressed as mean±standard deviation (SD), and statistical significance was shown as N $P>0.05$.

Supplementary figure.4

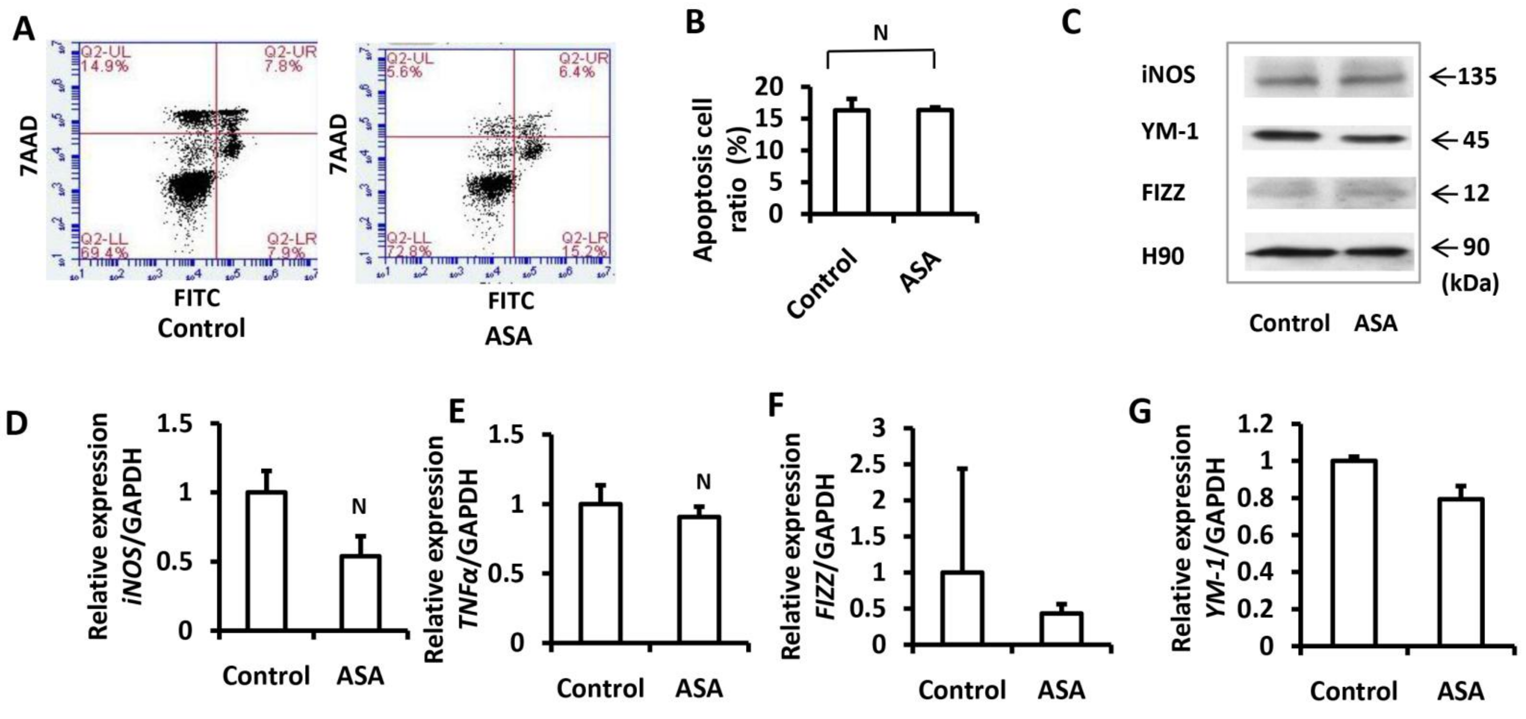

Supplementary Figure. 4. 200μg/ml ASA treatment didn't cause RAW264.7 cells apoptosis and had no effect on expression of iNOS, TNF-α, FIZZ or YM-1 in macrophage cells.
(A,B) ASA at 200μg/ml treated 36 hours on RAW264.7 had no effects on the apoptosis of cells. **(C)** Western blot showed the result that 200μg/ml ASA treatment on mouse peritoneal macrophages had no effects on the expression of iNOS, YM-1 or FIZZ. Full-length gels are presented in Supplementary Figure 11. **(D,E)** The result of PCR confirmed ASA at 200μg/ml had no effects on the total RNA expression of iNOS or TNF-α. **(F,G)** The result of PCR confirmed ASA at 200μg/ml had no effects on the total RNA expression of FIZZ or YM-1.
All results are representative of at least three independent experiments. Results were expressed as mean±standard deviation (SD), and statistical significance was shown as N $P>0.05$.

Supplementary figure.5

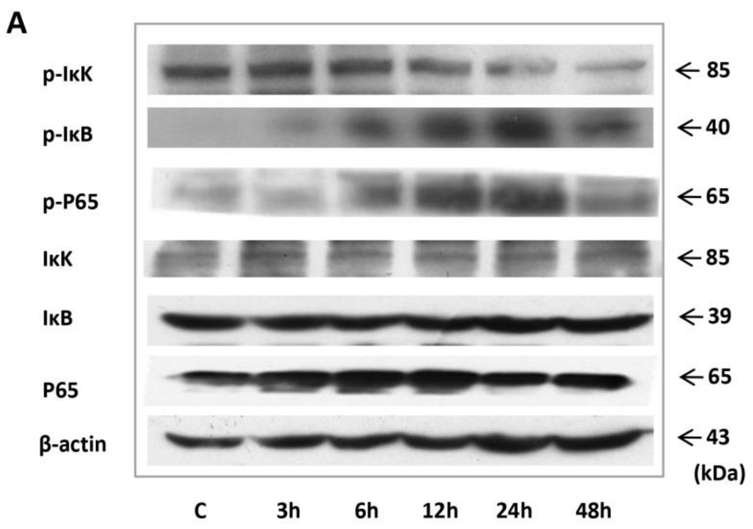

Supplementary Figure. 5. NF-κB pathway time course induced by LPS.
(A) NF-κB pathway time course showed that when RAW264.7 induced by LPS, the protein expression of p-IKK increased by the time of 3hours-6hours after inducement, and went down after 12hours. However, the protein expression of p-IKB and p-P65 increased from 6hours-24hours after LPS inducement, and went down by the 48hours after LPS inducement. Full-length gels are presented in Supplementary Figure 12.
All results are representative of at least three independent experiments.

Supplementary figure.6

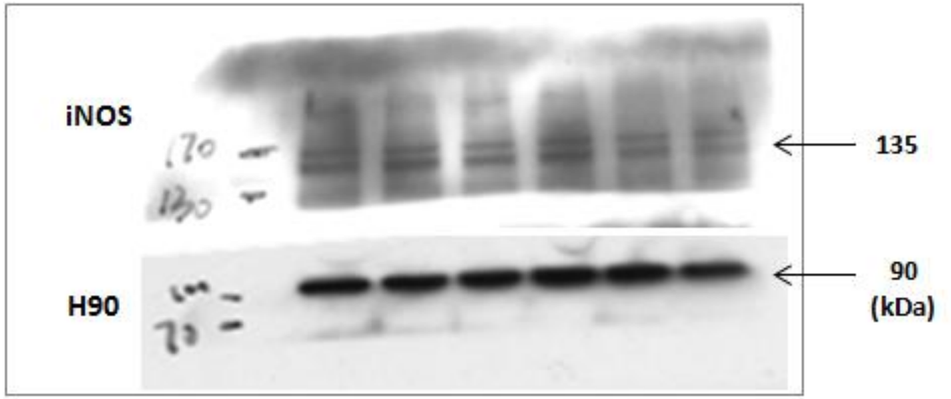

The full-length gels for the FIG.1C.

Supplementary figure.7

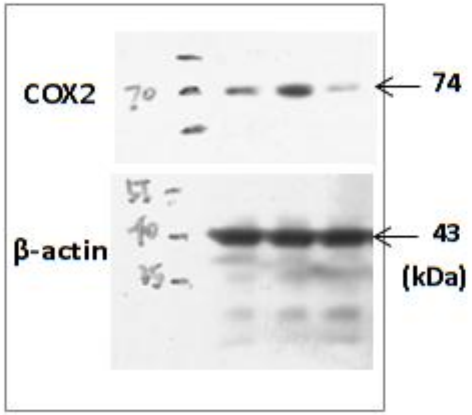

The full-length gels for the FIG.2A.

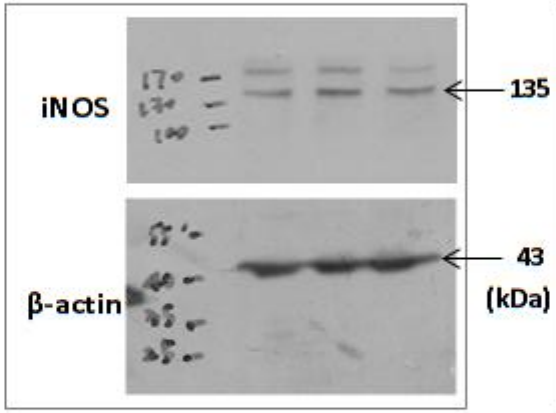

The full-length gels for the FIG.2D.

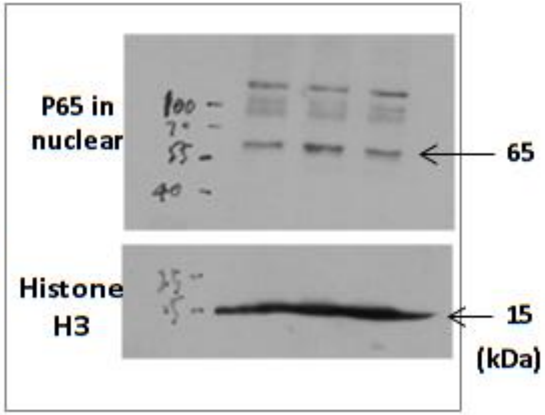

The full-length gels for the FIG.2F.

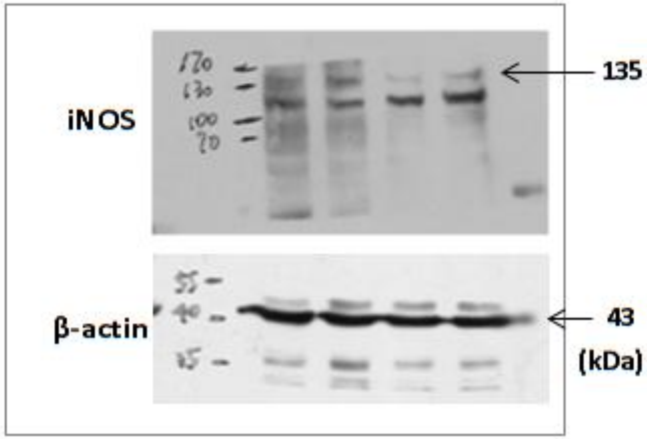

The full-length gels for the FIG.2G.

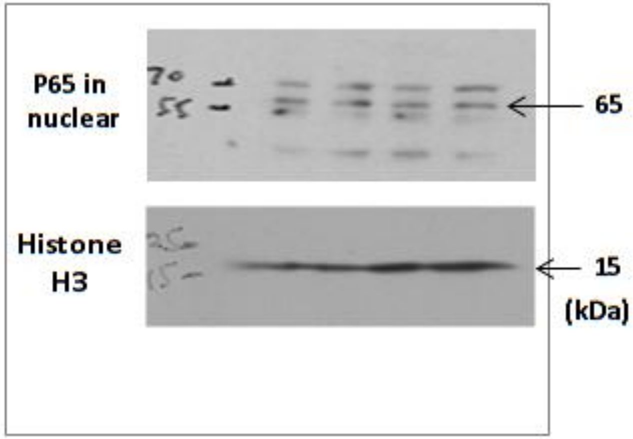

The full-length gels for the FIG.2H.

Supplementary figure.8

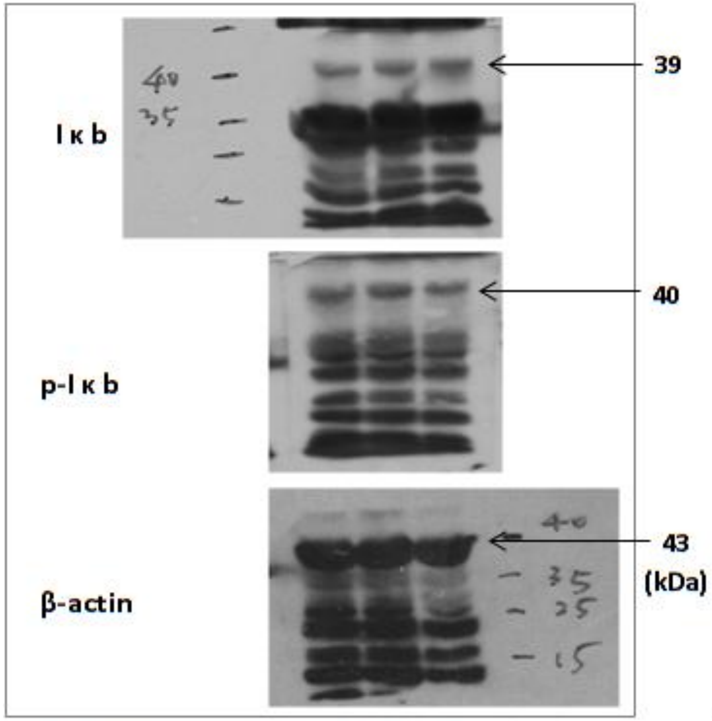

The full-length gels for the FIG.3A.

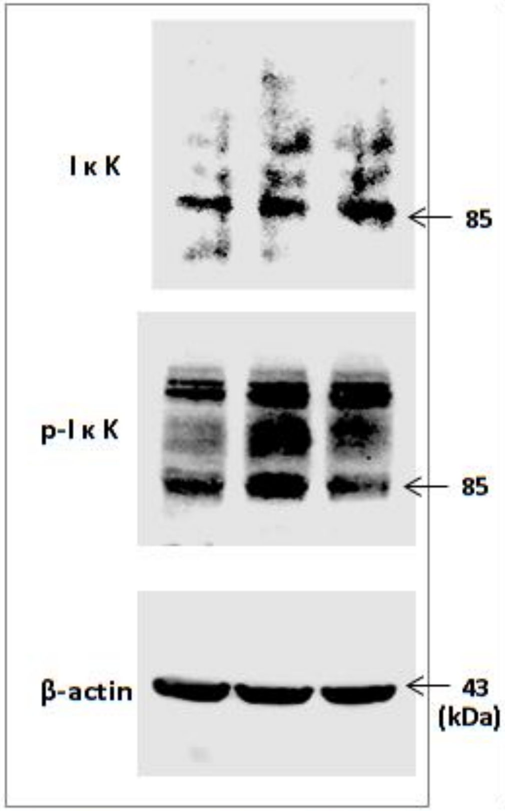

The full-length gels for the FIG.3A.

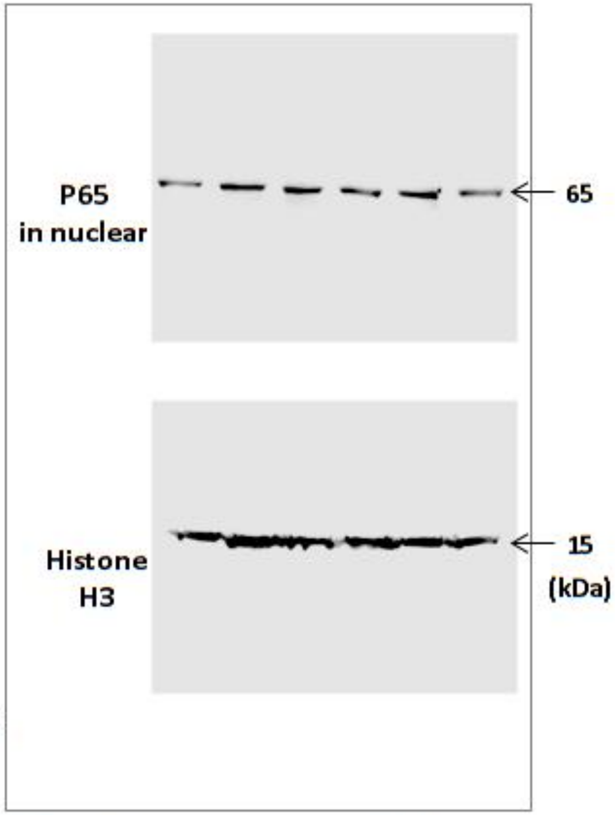

The full-length gels for the FIG.3A.

All digital images were exposed by Densitometry analysis performedusing FluorChemFC2 imaging system AlphaEaseFC program (Cell Biosciences, Santa Clara, CA, USA). The exposed time was 1 minute.

Supplementary figure.9

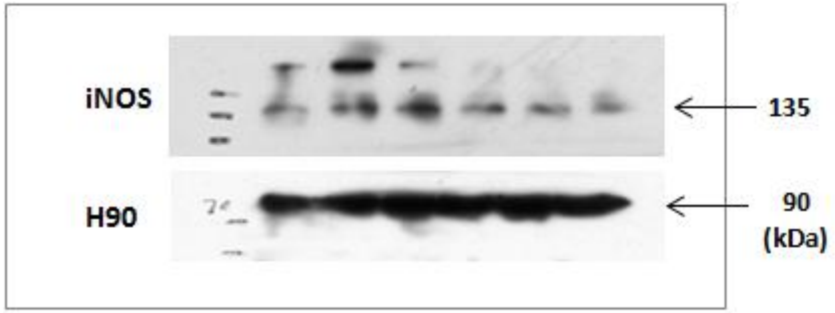

The full-length gels for the Suppl FIG.1C.

Supplementary figure.10

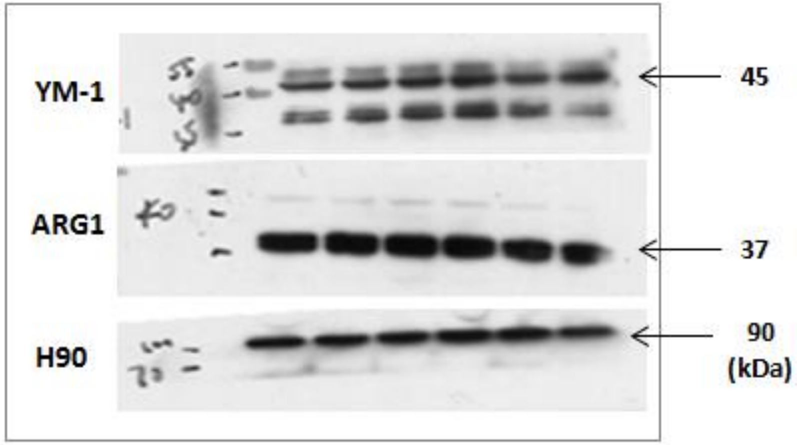

The full-length gels for the Suppl FIG.2B.

Supplementary figure.11

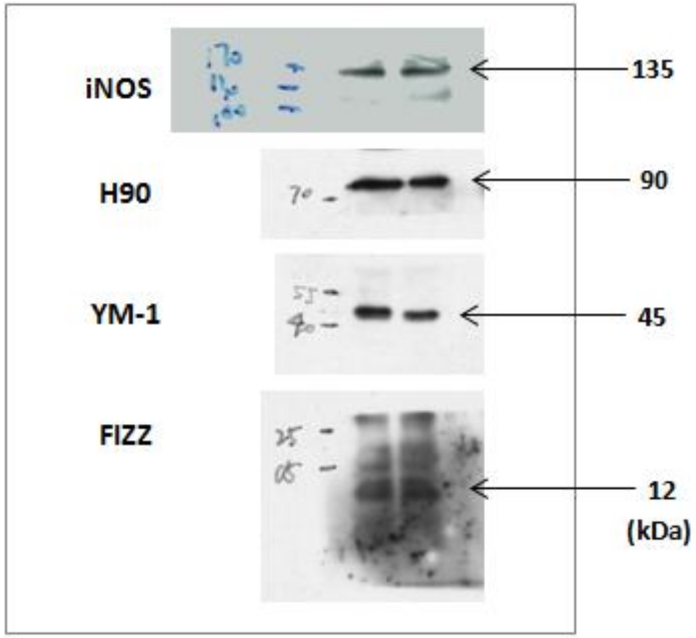

The full-length gels for the Suppl FIG.4C.

Supplementary figure.12

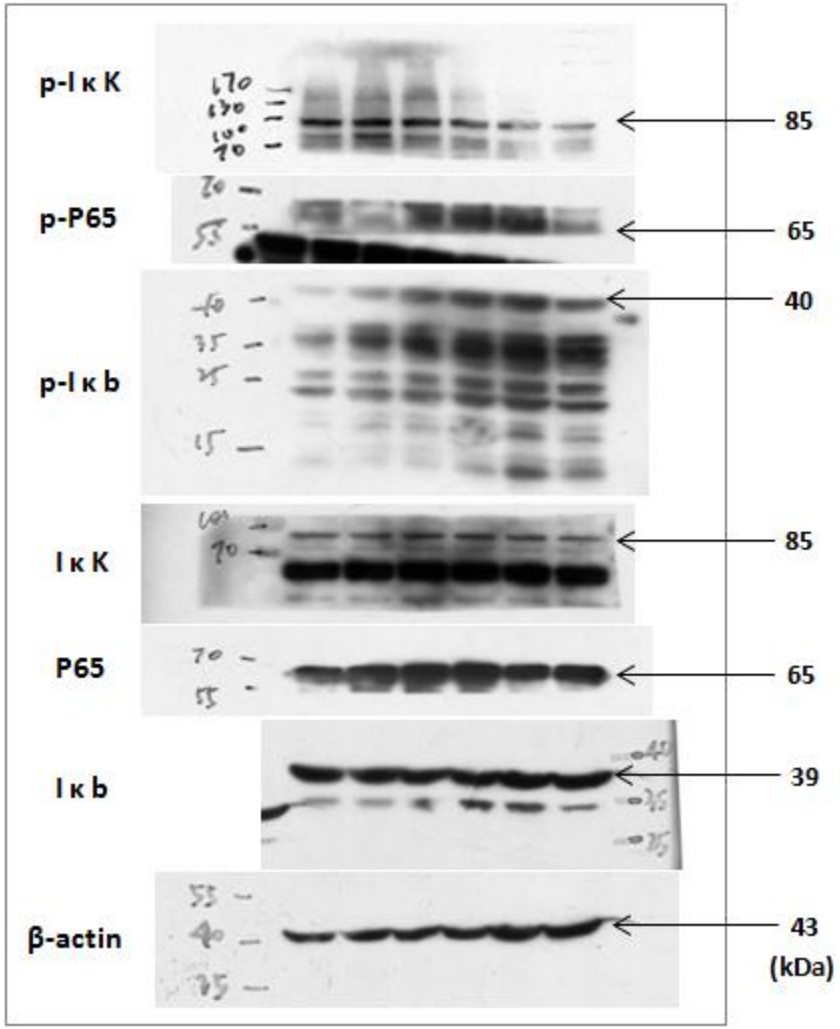

The full-length gels for the Suppl FIG.5A.

Table 1. Primers used in quantitative real-time PCR analysis

Target	Forward primer	Reverse primer
iNOS	5’-acccaaggtctacgttcagg-3’	5’-cgcacatctccgcaaatgta-3’
ARG1	5’-acaagacagggctcctttca-3’	5’-agcaagccaaggttaaagcc-3’
COX_2	5’-accgagtcgttctgccaata-3’	5’-ctcatgagtggaggacgtct-3’
YM-1	5’-ccatggccaagctcattctt-3’	5’-tcccttctattggcctgtcc-3’
FIZZ	5’-atgaacagatgggcctcctg-3’	5’-cccaagatccacaggcaaag-3’
TNFα	5’-ttccgaattcactggagcct-3’	5’-acctgaccactctccctttg-3’
